# The Work of the R.A.M.C.: Another Record of Gallant Deeds

**Published:** 1915-10-09

**Authors:** 


### THE WORK OF THE R.A.M.C.

### Another Record of Gallant Deeds.

### GALLANTRY AT HOOGE.

In the last article dealing with awards for bravery to members of the Royal Army Medical Corps and ambulance workers (The Hospital, September 25, p. 547), it was stated that the awards for the recent severe fighting in the Hooge district might soon be expected. Since that article was published the list has made its appearance in a special supplement of the *London Gazette*.

Dealing first with the Hooge awards, the Military Cross has been awarded to Lieut. William Brooks Keith, M.B., 1st Home Counties Field Ambulance, R.A.M.C. (Territorial Force), for conspicuous gallantry at Hooge. When a shell killed two stretcher-bearers who were bringing in a wounded officer, Lieut. Keith went out to assist, and under heavy fire brought the wounded officer into the dressing station. The official report adds that he has performed many acts of a like nature, and has consistently shown coolness and resource under fire.

Of the winners of the Distinguished Conduct Medal Corporal G. Gallagher, of the 16th Field Ambulance, R.A.M.C., is rewarded for conspicuous gallantry and devotion to duty from August 9 to 11 at Hooge in rallying stretcher-bearers and collecting the wounded under heavy fire. On the same occasion Sergeant J. H. Heap, of the 18th Field Ambulance, R.A.M.C., displayed conspicuous gallantry and devotion to duty in giving constant attention to the wounded under heavy fire. Private H. Meakins, of the R.A.M.C. (attached to the 2nd Battalion York and Lancaster Regiment), obtains his medal for conspicuous gallantry and good work throughout the campaign, notably at Hooge on August 9, when he gave great assistance to the medical officer under heavy shell fire, and when alone in the trenches worked hard all day tending the wounded and dressing their wounds.

The other awards deal with the campaign generally in Flanders and the Dardanelles. Captain Hugh Glencairn Monteith, R.A.M.C., and attached to the 2nd Battalion of the Duke of Cornwall's Light Infantry, is awarded the Distinguished Service Order for conspicuous gallantry and devotion to duty in picking up and attending to the wounded under heavy fire in the actions near St. Jean and Wieltje between April 23 and 27, when the casualties in the battalion to which he was attached were very heavy. Private W. Hughes and Private H. Price, of the East Lancashire Field Ambulance, R.A.M.C. (T.F.), are awarded the Distinguished Conduct Medal for gallantry on the Gallipoli Peninsula at the beginning of June. It is recorded that they continued their work throughout the fighting under machine-gun and shrapnel fire with a total disregard to danger. Of Private A. P. Inglis, of the 3rd Lowland Field Ambulance, R.A.M.C. (T.F.), who also receives the medal, it is recorded that, though himself wounded in the leg, and tired out by almost continuous duty for three days, he continued to attend to and carry back the wounded under shell and rifle fire.

### MILITARY CROSS FOR R.A.M.C. CAPTAIN.

The King has been pleased to confer the Military Cross on Captain Ernest Cotton Deane, of the Royal Army Medical Corps, who is attached to the 2nd Leicestershire Regiment, for conspicuous gallantry on August 22 near Fauquissart. The official report states that a standing patrol 120 yards in front of the British line was bombed by the enemy at about 10 p.m., the only notification being two loud bomb explosions. Captain Deane, without any knowledge of the enemy's strength, at once got over the parapet and ran by himself to the spot under rifle and machine-gun fire. Finding four wounded men he returned for stretchers and got them back into safety. It is added that this is not the first time that Captain Deane's gallantry under fire has been brought to notice.

